# Hydroxyapatite/collagen composite graft for posterior lumbar interbody fusion: a comparison with local bone graft

**DOI:** 10.1186/s13018-021-02798-4

**Published:** 2021-10-24

**Authors:** Toshitaka Yoshii, Motonori Hashimoto, Satoru Egawa, Takashi Hirai, Hiroyuki Inose, Atsushi Okawa

**Affiliations:** Department of Orthopedic Surgery, Tokyo Medical and Dental University, 1 Chome-5-45 Yushima, Bunkyo City, Tokyo, 113-8510 Japan

**Keywords:** Graft substitute, Hydroxyapatite with collagen, Local bone graft, Posterior lumbar interbody fusion, Fusion rate

## Abstract

**Background:**

Autologous bone has been used for posterior lumbar intervertebral fusion (PLIF). However, harvesting autologous bone graft is associated with donor site complications. We previously developed a hydroxyapatite/collagen (HAp/Col) composite as an osteoconductive artificial bone, characterized by having a highly porous structure with sponge‐like elasticity. This study aims to investigate the effectiveness of HAp/Col composite with bone marrow aspirate (BMA) as a graft substitute in PLIF for the treatment of lumbar spinal diseases.

**Methods:**

This study prospectively investigated patients who received one-level PLIF. For the interbody fusion, two titanium cages were inserted. On the one side of interbody space, HAp/Col composite incorporated with BMA filling the titanium cage was grafted. On the other side, local bone graft (LBG) harvested during decompressive laminotomy was grafted and then one-level instrumentation using pedicle screws was performed. The target levels were at L2/3 in 2 cases, L3/4 in 3 cases, L4/5 in 36 cases, and L5/S in 5 cases. We evaluated clinical symptoms and radiological outcomes of 46 patients and compared the fusion status of HAp/Col composite with that of LBG.

**Results:**

The 1-year postoperative CT evaluation demonstrated that, in the HAp/Col, a complete fusion was observed in 38 patients (82.6%), whereas in the LBG, a complete fusion was observed in 35 patients (76.1%). There were no statistical differences between the HAp/Col and LBG. In the HAp/Col, incomplete fusion was observed in five patients (10.9%) and non-fusion in two patients (4.3%), and in the LBG, incomplete fusion was observed in nine patients (19.6%) and non-fusion in two patients (4.3%). At 2 years after the surgery, complete fusion increased to 44 patients (95.7%) in the HAp/Col and 41 patients (89.1%) in the LBG. There were no significant differences in the clinical scores for lumbar spine between patients with fusion and non-fusion.

**Conclusions:**

The HAp/Col composite with BMA in the titanium cage can be effectively used as an alternative to conventional autologous LBG for intervertebral spinal fusion.

**Trial registration** University hospital Medical Information Network, UMIN000045010, July 30th, 2021, Retrospectively registered, https://www.umin.ac.jp/english/.

## Introduction

The autologous bone graft has been the gold standard for lumbar spinal fusion. Furthermore, the autologous bone harvested from the iliac crest or local bone from the spinous process and laminae has been used with cages to the intervertebral space for posterior lumbar intervertebral fusion (PLIF) [1, 2]. However, significant incidences of complications at the donor site associated with harvesting bone from the iliac crest, including donor site pain, hematoma, infections, fracture of the ilium, sensory disturbance, and cosmetic disability, were reported [3]. Moreover, local bone harvested from the spinous process and laminae is limited in amount, especially in revision surgery.

The hydroxyapatite/collagen (HAp/Col) composite was previously developed as an artificial bone substitute with distinctive characteristics [4–8]. HAp/Col composite is characterized by having a highly porous structure composed of collagen fibers with HAp nanocrystal deposits. HAp/Col composite has a sponge‐like elasticity and biocompatibility. A variety of graft substitutes have been increasingly studied for spinal fusion [9–11]. Most graft materials, including HAp/Col composite, have been proven both biocompatible and osteoconductive; however, few osteoinductive properties were observed in the materials themselves [12, 13]. Therefore, it is preferable to use biologically active tissue-inducing substances or cells with the ceramics.

Animal studies have proved that, due to the presence of osteoprogenitor cells and osteoinductive growth factors, bone marrow aspirate (BMA) exhibits osteopromotive properties when combined with porous ceramics [14, 15]. Besides, its osteogenic potential has also been shown in spinal fusion in both animal models and humans [14–18]. BMA has the clinical merits of being autologous and intraoperatively available, with harvesting possible through a simple and safe aspiration [15]. HAp/Col composite has a large surface area, and the nanoscale HA crystals enable it to absorb a large quantity of proteins and other molecules, such as drugs and ions [4]. Therefore, BMA integrated into the HAp/Col composite was used, and the graft in a titanium cage was implanted for the intervertebral spinal fusion.

In this study, we investigated the effectiveness of HAp/Col composite for spinal fusion using a “side-by-side” comparison model. The new HAp/Col composite incorporated with BMA filling the titanium cage was grafted on one side of the interbody space. On the other side, autologous local bone graft (LBG) harvested during decompressive laminotomy was grafted, and then, one-level instrumentation using pedicle screws was performed. We evaluated and compared the fusion status of both HAp/Col composite and LBG.

## Methods

This study was approved by the institutional review board. This study prospectively investigated 50 consecutive patients who underwent a 1-level PLIF from March 2014 to August 2016. Lumbar spinal canal stenosis with single-level segmental instability was diagnosed in 20 patients, 29 presented with degenerative spondylolisthesis, and 1 had isthmic spondylolisthesis. The patients underwent decompression laminotomy and pedicle screw instrumented PLIF. The patients who had a history of previous lumbar spine surgery, spinal infection, trauma, and multilevel spinal fusion were excluded from the study.

### Surgical techniques and preparation of the implants

The patients were placed in a prone position, and decompressive laminotomy was performed to remove the spinous process and lamina in the region of spinal canal stenosis under general anesthesia. To access the intervertebral disc, the facet joints were removed. The local bone chips extracted during laminotomy were morselized and collected by meticulously removing the covering soft tissues; these morselized bone chips were used as a graft on the control side. Then, the intervertebral disc was removed and the endplate was curetted. After the adequate size of cage was determined using a trial cage, one cage was filled with HAp/Col composite. Another cage was filled with LBG. We used the same-sized two titanium cages for the intervertebral space, with HAp/Col composite grafted on the one side and LBG grafted on the other side.

**Fig. 1:**
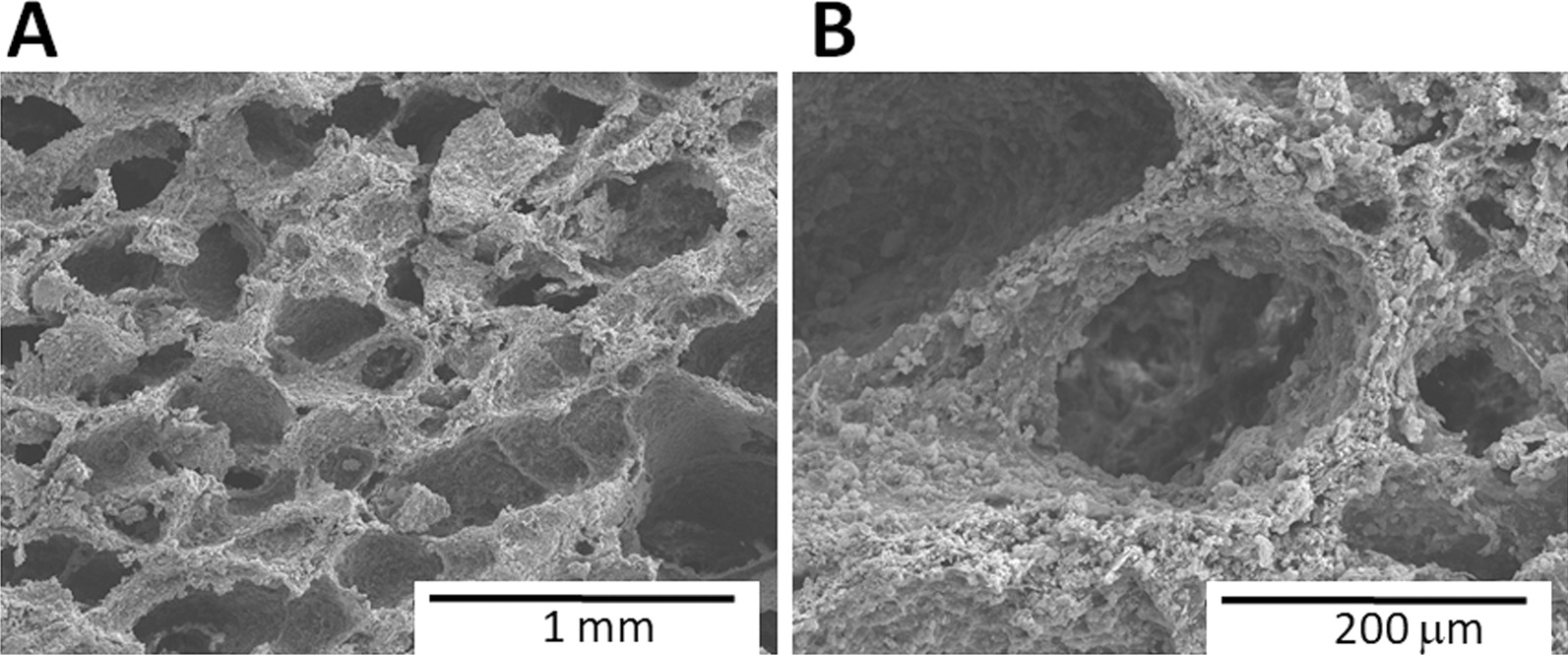
Scanning electron microscopy (SEM) demonstrates a highly porous structure of the hydroxyapatite/collagen composite (pore size: 100–500 μm, porosity: 95%) (**A**). High magnification demonstrates macropores and micropores (**B**)

We used scanning electron microscopy (SEM) to demonstrate the morphology of the composite HA. Also, a Hitachi S-24300 scanning electron microscope was used to acquire images (Fig. 1). The pore size and porosity of the porous HAp/Col composite were 100–500 μm and 95%, respectively.

Approximately, 3 mL of BMA was aspirated from the posterior iliac crest with 11-gauge Jamshidi bone marrow biopsy/aspiration needle. The harvested BMA was immediately integrated into the HAp/Col composite. Then, we inserted HAp/Col composite and LBG with titanium cage (Telamon®, Medtronic; O.I.C. Titanium ®, Stryker) into the intervertebral space. After the insertion of the cages, we used the transpedicular screw/rod instrumentation to perform posterior fixation. On postoperative day 2, all patients were encouraged to begin walking. Furthermore, a soft brace was prescribed for 3 months postoperatively.

### Outcomes

The clinical outcomes were assessed using the scoring system proposed by the Japanese Orthopedic Association (JOA) score [19]. We calculated the recovery rate of JOA score according to the following formula: the recovery rate (%) = [(postoperative score − preoperative score)/(29 − preoperative score)] × 100.

We evaluated and recorded the fusion status in this study and performed radiographic examinations including pre- and postoperative plain and functional X-rays. We also measured the segmental angles of the operated vertebral levels in a neutral position, as well as in extension and flexion.

The postoperative flexion/extension X-ray shows that angular motion ≥ 3° was recorded as segmental instability. Cage subsidence was defined as a vertical migration of the cage into the vertebral body by ≥ 2 mm. Screw loosening was defined by a radiolucent zone surrounding the screw thicker than 1 mm. We performed CT evaluation with coronal/sagittal reconstruction before surgery and after surgery at 1 year.

**Fig. 2:**
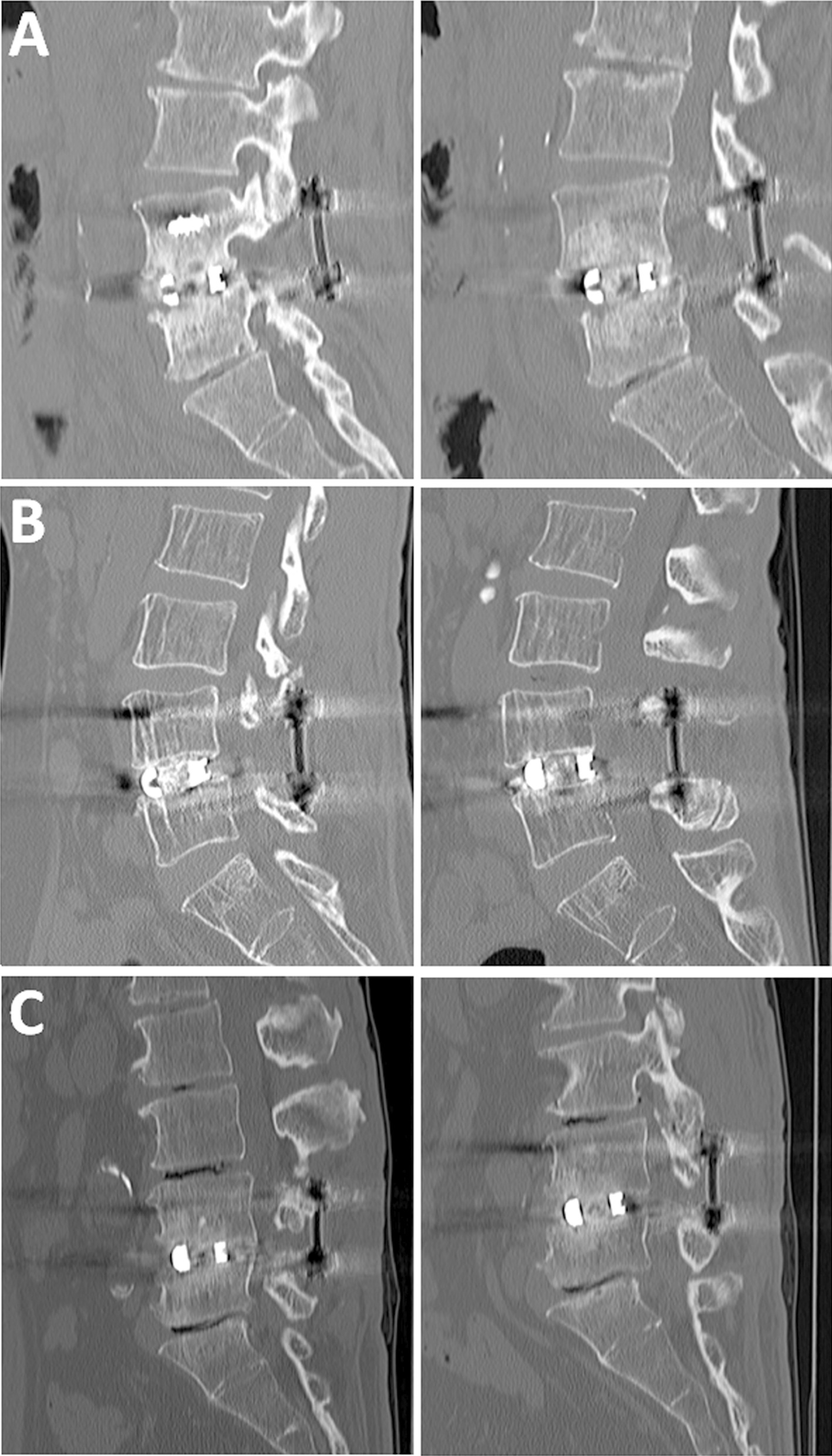
Fusion status evaluated by computed tomography (CT) at 1 year postoperatively. Left lane: HAP/Col graft, Right lane: Local bone graft. **A** Non-fusion: no obvious bony bridging was observed. Clear radiolucent line between the graft and endplate or cyst formation was observed. **B** Incomplete fusion: bony bridging was partly observed, but not completed. The endplates were still visible. **C** Complete fusion: a continuous bony bridging was obviously observed

The assessment of the fusion status was performed by two independent spine surgeons in a blinded manner. Based on the reconstructed CT pictures, we simply defined the fusion status as follows: (1) Non-fusion: no bony bridging was observed (Fig. 2A). (2) Incomplete fusion: bony bridging was partly observed, but not completed (Fig. 2B). (3) Complete fusion: a continuous bony bridging was obviously observed (Fig. 2C). We performed CT evaluation for the patients who did not show complete fusion at 2 years after the surgery.

The statistical analysis was carried out using the paired *t*-test and Chi-square test. The confidence interval was set at 95% and the significance level at *p* < 0.05. The κ coefficient (Cohen) was used to evaluate the interobserver agreement in the fusion status scoring. Of the 50 patients included, four patients were excluded (3 with degenerative spondylolisthesis, and 1 suffering from lumbar spinal canal stenosis) because of the absence of CT evaluation (2 patients) and because of other diseases (2 patients).

## Results

In this study, all the patients tolerated the surgical procedure well. Forty-six patients (92.0%) [13 males and 33 females, with an average age of 71.3 years (range 51–83)] completed a 1-year follow-up evaluation (Table 1). The target levels were at L2/3 in 2 cases, L3/4 in 3 cases, L4/5 in 36 cases, and L5/S in five cases.

**Table 1:** Demographics

1-level PLIF case, No	46
Male/female	13/33
Age	71.3 (51–83)
Diseases	21 (3.7%)
degenerative spondylolisthesis	26
Lumbar spinal canal stenosis	19
isthmic spondylolisthesis	1
Level	
L2/3	2
L3/4	3
L4/5	36
L5/S	5
Pre JOA score (points)	15.8 ± 3.0
Post JOA score (1 year)	25.3 ± 2.7
Recovery rate (%)	71.1 ± 21.5
Pre-local lordosis (degrees)	12.0 ± 7.7
Post-local lordosis (1 year)	12.9 ± 7.0

**Fig. 3:**
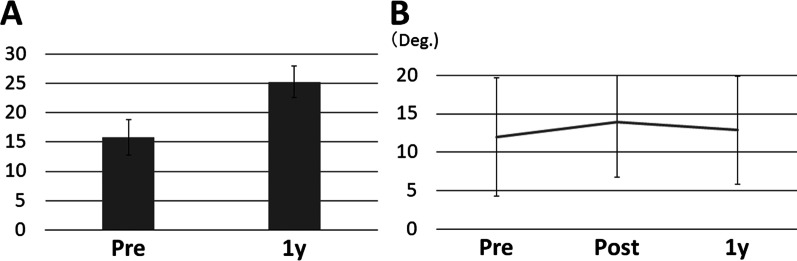
**A** Preoperative and postoperative (1 year) neurological scores (JOA score). **B** Change of local lordosis of the fused segment before and after surgery and at 1 year postoperatively

After the surgery, the neurological symptoms improved. The preoperative average JOA score was 15.8 ± 3.0 (range 9–23) points, and the postoperative JOA score improved to 25.3 ± 2.7 (range 21–29). The average recovery rate was 71.1% (Fig. 3). Of the 50 cases initially included in this study, incidental dural tear occurred in 1 case, which was repaired intraoperatively. A femoral neck fracture occurred in 1 case during the follow-up, and a hip surgery was performed on the patient.

In the fusion status evaluation using a reconstruction CT scan, the interobserver reliability revealed a good agreement (Cohen’s Kappa score: 0.75). CT evaluation at 1 year postoperatively demonstrated that, in the HAp/Col, a complete fusion was observed in 38 patients (82.6%), whereas in the LBG, it was observed in 35 patients (76.1%). There were no statistical differences between the HAp/Col and LBG (Table 2). On the other hand, in patients who did not show complete fusion at 1 year, in the HAp/Col, incomplete fusion was observed in five patients (10.9%) and non-fusion in two patients (4.3%), and in the LBG, incomplete fusion was observed in nine patients (19.6%) and non-fusion in two patients (4.3%). A complete fusion in the HAp/Col increased to 44 patients (95.7%) and 41 patients (89.1%) in the LBG at 2 years after the surgery.

**Table 2:** Fusion status by CT evaluation

Fusion status	HAp/Col (*n* = 46)	LBG (*n* = 46)	*P* value
1 year			
Non-fusion	2 (4.3%)	2 (4.3%)	0.32
Incomplete	5 (10.9%)	9 (19.6%)	
Complete	38 (82.6%)	35 (76.1%)	
2 years			
Non-fusion	0 (0%)	0 (0%)	0.24
Incomplete	2 (10.9%)	5 (19.6%)	
Complete	44 (95.7%)	41 (89.1%)	

In the dynamic X-rays, we observed that only 1 case (2.2%) exhibited obvious segmental instability (> 3° of angular motion) at 1 year after surgery. The subsidence of the cage was found in 12 patients (26.1%), whereas no patients showed cage dislodgement. There was apparent screw loosening in 3 cases (6.5%). The local alignment (Cobb angle) was enhanced after surgery, but reduced to the preoperative level at 1 year (Fig. 3).

We further compared the group with complete fusion at both cages and the group with incomplete/non-fusion at either of the cages. There were no significant differences in baseline demographics and surgical outcomes between the two groups, but the age of patients in the group with incomplete/non-fusion tended to be higher (*p* = 0.08) (Table 3).

**Table 3:** Cases with complete fusion vs incomplete/non-fusion

	Complete fusion (*n* = 32)	Incomplete/non-(*n* = 14)	*P* value
Male/female	8/24	4/10	0.80
Age	70.2 ± 8.3	73.8 ± 5.2	0.08
Diseases			0.36
Degenerative spondylolisthesis	16	10	
Lumbar spinal canal stenosis	15	4	
Isthmic spondylolisthesis	1	0	
Levels			0.89
L2/3	1	1	
L3/4	2	1	
L4/5	25	11	
L5/S	4	1	
Pre-JOA score	16.0 ± 3.0	15.1 ± 3.1	0.36
Post-JOA score (1 year)	25.2 ± 2.9	25.4 ± 2.4	0.79
Recovery rate (%)	70.5 ± 22.3	72.5 ± 20.3	0.78
Pre-local lordosis	12.1 ± 8.2	11.7 ± 6.7	0.88
Post-local lordosis (1 year)	13.4 ± 11.8	11.8 ± 5.6	0.49
Subsidence (+)	9 (28.1%)	4 (28.6%)	0.98

## Discussion

Lumbar spine fusion has been a common practice for treating degenerative spinal diseases. The ideal bone graft materials for spinal fusion require osteoinductivity, osteoconductivity, and excellent mechanical properties. Iliac crest bone graft (ICBG) has been traditionally used because it possesses all the characteristics required for an ideal bone graft, which include trabecular structure as an osteoconductive scaffold, osteoinductive bone morphogenetic proteins, and osteogenic cells. However, the significant complications associated with harvesting ICBG have encouraged an ongoing interest in finding alternative graft materials [3].

Calcium-based synthetic materials have been developed as bone graft extenders and/or substitutes. The HAp/Col composite consists of nanoscale hydroxyapatite (80 w/w %) and porcine skin-derived atelocollagen (20 w/w %), and its nanostructure is similar to that of natural bone [4, 6]. The highly porous structure of HAp/Col composite can achieve excellent osteoconductivity. In previous studies using various animal models, high osteoconductivity and bioabsorbability of HAp/Col composite were demonstrated [4, 6, 8]. Furthermore, once the porous body of HAp/Col composite is wetted, it becomes elastic like a sponge and is thus easy to implant into bone defects of various shapes and also be inserted in the PLIF cage.

In the present study, we examined the HAp/Col composite integrated with BMA as a graft material implanted in the intervertebral space with the PLIF cage. It is known that the BMA possesses osteogenic potential because it contains both osteoprogenitor cells and bone morphogenetic growth factors [20, 21]. Connolly et al. reported the osteopromotive property of BMA when injecting autologous marrow into tibial nonunions [22]. Moreover, the use of BMA and osteoconductive scaffolds in posterolateral lumbar fusion was reported in several clinical studies. Epstein et al. reported the efficacy of BMA with TCP/type I collagen synthetic composite and local autograft as a bone expander in posterolateral spinal fusion [18]. Neen et al. in their prospective study used BMA with hydroxyapatite/type I collagen synthetic matrix in posterolateral lumbar fusion and showed equivalent outcomes when compared with autologous ICBG [23].

In this study, we investigated the efficacy of the HAp/Col composite with BMA filled in a titanium cage in the posterior approached interbody fusion surgery. There are two major types of cages that are currently used in PLIF: titanium and polyetheretherketone (PEEK) cage. We used titanium cages in this study, because the fusion rate of metal cages is reported to be superior to that of PEEK cages in PLIF [24]. As described previously, HAp/Col composite has a sponge-like mechanical property; thus, it is very easily integrated with BMA and packed inside the titanium cage. A previous study also examined this material in the lumbar lateral interbody fusion cage and proved the clinical usefulness of the HAp/Col composite [25]. In this study, we applied the HAp/Col composite in 50 patients and it showed excellent bioavailability; no significant adverse events regarding the HAp/Col composite were observed.

We evaluated the intervertebral spinal fusion with Hap/Col in a titanium cage by using reconstruction CT, which showed a good interobserver reliability (Kappa score: 0.75). Evaluation based on bony continuity on reconstructed CT is considered as the “golden standard” to monitor the progression of an interbody fusion [26]. Although dynamic lateral flexion and extension radiographs have been used, calculating subtle degrees of motion on flexion/extension films is irreproducible. In a study using sheep, Sandhu et al. showed that only 33% were judged fused on histological pictures in cases that were judged as radiographic fusion by dynamic X-rays [27]. On the other hand, Cunningham et al. [28], in their study using cages in sheep spine, showed a 100% correlation between CT and histological images. Thus, in this study, we used simple fusion criteria based on bony bridging on the reconstructed CT images, which is considered to have substantial accuracy for assessing the interbody fusion.

We compared the HAp/Col composite with BMA with the autologous local bone graft (LBG), which is commonly used for lumbar interbody fusion. The HAp/Col composite showed a tendency to higher complete fusion rate (82.6% and 95.7%, at 1 year and 2 years, respectively), compared with the rate of LBG (76.1% and 89.1%, at 1 year and 2 years, respectively). A previous systematic review has reported that the fusion rate of single-level PLIF using ICBG or LBG averaged 89.5% at 2 years postoperatively [29]. The HAp/Col composite with BMA in a titanium cage demonstrated a comparable fusion rate to those previously reported using an autologous bone graft. A previous study has reported that PLIF using another clinically available calcium phosphate, β-TCP with BMA, showed a non-fusion rate of 38.6% at 1 year [30]. The reason for this high non-fusion rate may be explained by the material adaptability to the vertebral endplates above and below the interbody space. Because the β-TCP has no elastic property, the fitting of the material to the vertebral endplates is not good enough, which results in a lower interface area between the bone and material. On the other hand, the merit of using HAp/Col composite is its excellent capability of fitting to the endplate, which enhances the interface activity and bone formation in the material. Thus, HAp/Col composite would be a more suitable filling material in cages for PLIF. In addition, because the stiff mechanical property of titanium cages can cause shaving to the endplate, which may cause a high rate of subsidence, this may also enhance recruitment of the progenitor cells to the material.

There are some limitations in our study. (1) We used a “side-by-side” comparison model to compare the HAp/Col composite and LBG as a filling material in cages for PLIF. In this model, the fusion or lack of fusion on the one side may influence the progress of fusion on the opposite side. (2) We used local laminectomy bone as a control instead of using ICBG. However, the LBG has recently been shown to achieve an equivalent fusion rate in one-level fusion. (3) Radiological changes at the adjacent levels were not evaluated. It is known that the fusion of spinal segments leads to degenerative changes in the mobile segments above or below the fused spine, including sacroiliac joint [31]. Further long-term observation is needed to confirm the clinical efficacy of PLIF using this HAp/Col composite. Despite these limitations, this study has demonstrated that the graft of HAp/Col composite with BMA in a titanium cage facilitated bony fusion in PLIF.

## Conclusion

The HAp/Col composite with BMA demonstrated an excellent fusion rate, which is comparable to that of LBG. This hybrid construct can be effectively used as an alternative to conventional autologous LBG for intervertebral spinal fusion.

## Data Availability

The datasets generated and/or analyzed during the current study are available from the corresponding author on reasonable request.
